# Development of a GA-Fuzzy-Immune PID Controller with Incomplete Derivation for Robot Dexterous Hand

**DOI:** 10.1155/2014/564137

**Published:** 2014-07-06

**Authors:** Xin-hua Liu, Xiao-hu Chen, Xian-hua Zheng, Sheng-peng Li, Zhong-bin Wang

**Affiliations:** ^1^School of Mechanical and Electrical Engineering, China University of Mining and Technology, Xuzhou 221116, China; ^2^Xuyi Mine Equipment and Materials R&D Center, China University of Mining and Technology, Huai'an 211700, China

## Abstract

In order to improve the performance of robot dexterous hand, a controller based on GA-fuzzy-immune PID was designed. The control system of a robot dexterous hand and mathematical model of an index finger were presented. Moreover, immune mechanism was applied to the controller design and an improved approach through integration of GA and fuzzy inference was proposed to realize parameters' optimization. Finally, a simulation example was provided and the designed controller was proved ideal.

## 1. Introduction

In the past few years, massive research is committed to study the anthropomorphic robot hands with dexterous manipulation abilities. As an important tool to improve the intelligence and manipulation levels of robots, multi-DOF and multisensory robot dexterous hand has become one of the most promising researches in robot field [1, 2]. The robot dexterous hand could distinguish objects with different materials and shapes and snatch them successfully through the control system. Therefore, the robustness and control accuracy of a control system would play an important role in evaluating the performance of a robot dexterous hand [3].

Nowadays, robot dexterous hands have been used in many fields such as industry field, agriculture field, service field, and medical rehabilitation field. However, most of them have some common disadvantages such as slow response, poor flexibility, weak anti-interference ability, and poor controllability [4, 5]. To the best of our knowledge, the problem of robust and intelligent control for a robot dexterous hand has almost not been dealt with. Based on our past researches on robot dexterous hands and control methods, this paper tries to tackle this problem.

Bearing the above observation in mind, a GA-fuzzy-immune PID (genetic algorithm-fuzzy- immune proportion-integration-differentiation) controller with incomplete derivation for robot dexterous hand is developed and the rest of this paper is organized as follows. In Section 2, some related works are outlined based on the literatures. The control system of a robot dexterous hand and mathematical model of an index finger are presented in Section 3. In Section 4, the GA-fuzzy-immune PID controller is designed and some improvements are proposed.  Section 5 provides a simulation example to verify the feasibility and efficiency of proposed controller. Our conclusions and future works are summarized in Section 6.

## 2. Literature Review

Recent publications relevant to this paper are mainly concerned with three research streams: robot dexterous hand control methods, PID control methods, and fuzzy-immunity feedback control methods. In this section, we try to summarize the relevant literatures.

### 2.1. Robot Dexterous Hand Control Methods

For the robot dexterous hand control methods, many researchers had worked on the problem and proposed different solutions since the last decades. As early as in 1962, a robot dexterous hand named after Belgrade was designed by Tomovic and Boni based on the most advanced control theory, which was considered to be the real significance dexterous hand [6]. Nowadays, with the development of computer technology, microelectronics technology, and advanced control theory, robot dexterous hand has entered a new period. Jafarov et al. [7] took both sliding and stability issues into account to present an augmented sliding surface design for robot hand. In [8], a new variable structure PID controller design approach was considered for the tracking stabilization of robot motion. Atia [9] designed a new nonlinear PID sliding mode controller for set-point control of robot hand, which ensured that the error tended to zero asymptotically if there was no disturbance applied to the robot dynamics. Chen et al. [10] presented two types of adaptive control program combining conventional computed-torque control and different fuzzy compensators for the robust tracking control of robotic manipulators with structured and unstructured uncertainties. In [11], a model-free recurrent fuzzy neural network (RFNN) control system for robot hand was proposed to approximate the ideal backstepping control law, which was further proved stable by the Lyapunov stability analysis. By combining feedback linearization with Lyapunov's second method and genetic algorithm, Hassanzadeh et al. [12] designed a robust controller with performance tuning for robot hand, and the stability and robust performance of proposed controller were verified through a four-bar linkage robot simulation. In [13], two fault-tolerant control strategies for robot hand were implemented based on output-feedback *H*_*∞*_ controller and experimental results illustrated that the improvements were feasible and efficient.

### 2.2. PID Control Methods

As one of the earliest control strategies, PID control has been developed to deal with more complex control problems due to the advantages of simple description, high dependability, strong robustness, and so forth. Han [14] proposed a nonlinear PID controller with the capability of auto-disturbance-rejection control and combination of differentiator and extended state observer, and transition process overcame the disturbance effectively and improved the control performance. Besides, Su et al. [15] applied the method of Han proposed for controlling of manipulator successfully. Gundes and Ozguler [16] investigated the problem of closed-loop stabilization using PID controller for MIMO plants to show the existence of stabilizing PID controllers for MIMO plants. Alvarez-Ramirez et al. [17] addressed the position regulation problem of robot manipulators under control input constraints and experiment results showed that the saturated linear PID control was semiglobally asymptotically stable. Oliveira et al. [18] used Hermite-Biehler theorem to establish results on the design of PID controllers for a class of time delay systems. Ziegler and Nichols [19] proposed the most well-known Ziegler and Nichols tuning formula for PID parameter tuning. Chen and Huang [20] presented a method for regulating PID parameters on line automatically with neural net algorithm. Neurofuzzy controller and genetic-fuzzy controller for second-order control systems were presented to improve the performance of conventional PID and fuzzy controller [21–23]. Genetic-fuzzy controller was applied in the drum boiler simulated dynamics to improve the control speed and precision [24]. Moreover, further improvements for neurofuzzy controller and genetic-fuzzy controller were carried out by genetic-neurofuzzy arithmetic [25–27]. Kim et al. [28] achieved automatic tuning of PID parameters through integration of taking *H*_*∞*_ as performance index and particle swarm optimization algorithm. Juang and Lu [29] proposed power-system load-frequency control by fuzzy-PI controller and simulations on a multiarea interconnected power system with different kinds of perturbations were performed to verify the performance of the proposed approach. Lu et al. [30] proposed an evolutionary fuzzy lead-lag control approach for coordinated control of flexible AC transmission system devices in a multimachine power system. Tang et al. [31] put forward a new method integrated with genetic algorithm and fuzzy distance to tune parameters. Zheng et al. [32] applied linear matrix inequalities (LMIs) in PID controller and a numerical example validated the stability of the closed-loop systems, *H*_2_ or *H*_*∞*_ performance specifications, or maximum output control requirement, respectively.

### 2.3. Fuzzy Immunity Feedback Control Methods

Back to 1986, Farmer et al. [33] suggested a dynamic model of an immune system based on immune network theory firstly and discussed the links between an immune system and other artificial intelligence methods. Xin et al. [34] designed a fuzzy-immune-PD-type control algorithm for trajectory tracking based on dynamics nonlinearities of robot manipulator, and experimental results showed that the control scheme had better tracking precision, stronger robustness, and superior control performance to conventional PD controller. Lei and Ren-hou [35] proposed a fuzzy immune algorithm to design a classification system, and the results of comparison with other classification schemes demonstrated the effectiveness of the proposed immune algorithm. Wang et al. [36] designed a fuzzy-immune-PID control system based on a mutative scale chaos optimization method to avoid a mass of tuning parameters work in the progress of design. An immune-fuzzy sliding mode controller (FISMC) was presented not only eliminating the synchronous reluctance motor system uncertainty but also overcoming the drawback of sign function and sat function [37]. Chang et al. [38] presented an effective procedure based on fuzzy logic and immune algorithm for the placement and sizing of shunt capacitor banks in a distorted power network. Kuo et al. [39] proposed an artificial immune system (AIS) based on fuzzy neural network (FNN) to avoid falling into the local optimum and improve the learning capability.

### 2.4. Discussion

However, although many approaches for robot dexterous hand have been proposed in above literatures, they have some common disadvantages summarized as follows. Firstly, some proposed controllers for self-adaption robot dexterous hand need to calculate the inverse of Jacobian matrix, but it is difficult to obtain and would consume much time. Secondly, due to the frictional disturbances at joints and external disturbance of payload, it is difficult to design a faster response, less overshoot, and satisfactory robust stability control system. Thirdly, the performance of some methods is actually related to specific weights, which is difficult to obtain. Finally, because of inherent deficiencies of some methods, it is easy to produce premature convergence.

In order to solve the above problems, a PID position controller based on immunity feedback control theory, fuzzy inference, and improved genetic algorithm is designed. A simulation example is provided and experiment results show that the proposed controller can achieve shorter adjust time, better rapidity, and higher steady-state precision than traditional PID position controller.

## 3. Robot Dexterous Hand

### 3.1. Robot Dexterous Hand Control System

**Figure 1:**
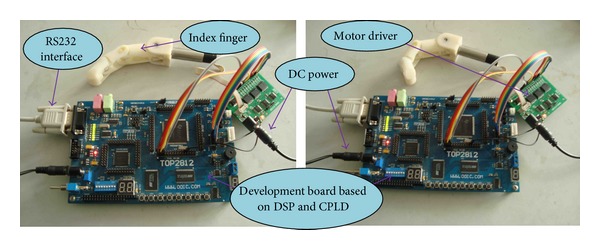
The control circuit board of robot dexterous hand and the index finger.

A dexterous hand (named after ABS-I) has been developed in our laboratory, which is made by the reinforced acrylonitrile butadiene styrene copolymers (ABS) in a 3D printer. It is composed of DC servo motors, cup-type planetary gear reducers, sensors, IE2-400 encoders, complicated programmable logic device (CPLD), and digital signal processor (DSP) unit.  Figure 1 shows the control circuit board of robot dexterous hand and the index finger.

**Figure 2:**
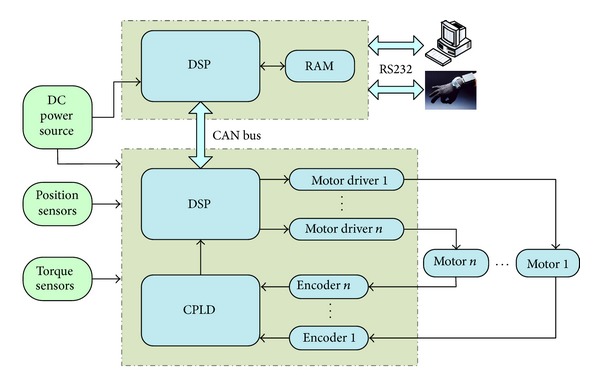
The robot dexterous hand control system.

The hierarchical control strategy adopted by the dexterous hand control system takes perfect purpose in practice. Feedback data glove or personal computer as the upper microcomputer communicates with bottom-level block through serial communication interface (SCI). The top-level block is responsible for the signal processing of upper microcomputer and the communicating with bottom-level block. The bottom-level block consists of DSP-CPLD servo controller, SCI circuit, motor driver, and so forth, and it is responsible for the signal processing of torque sensors, position sensors, and magnetoelectric encoders. Moreover, it is responsible for controlling the pulses and directing signals to drive servo motors. The dexterous hand control system can be shown as in Figure 2.

### 3.2. Mathematical Model for the Index Finger

Taking the single multijoint finger as an example, the equation of DC servo drive motor on armature loop [40] can be introduced as follows:

(1) $$\begin{array}{c} U_{a}=R_{a}i_{a}+L_{a}{\dot{i}}_{a}+E_{a}, \end{array}$$

where *U*_*a*_ is the armature control voltage, *R*_*a*_ is the armature resistance, *i*_*a*_ is the instantaneous current in coil, *L*_*a*_ is the armature inductance, *E*_*a*_ is the back electromotive force produced by coil, *E*_*a*_ = *K*_*e*_ *dθ*/*dt*, *θ* is the motor angle, and *K*_*e*_ is the voltage feedback coefficient.

Based on torque equations [41] of DC servo motor, the torque equation of single multijoint finger can be expressed as follows:

(2) Te=Jmθ¨+Bmθ˙+TL,

(3) Te=KTia,

where *T*_*e*_ is drive torque of motor, *K*_*T*_ is the motor moment coefficient, *J*_*m*_ is the equivalent moment of inertia of motor, *B*_*m*_ is the viscosity damp coefficient of motor, *T*_*L*_ is the load torque, $T_{L}=J_{L}{\ddot{\theta }}_{L}+B_{L}{\dot{\theta }}_{L}$, *J*_*L*_ is the equivalent moment of inertia of the finger, *B*_*L*_ is the viscosity damp coefficient of the finger, and *θ*_*L*_ is the distal phalanx. Among them, the relationship between *θ* and *θ*_*L*_ is expressed as *θ* = *θ*_*L*_ *N*, where *N* is the general transmission ratio.

In the synthesis, ignoring reducer clearance and transmission error of mechanism, the position transfer function of control voltage and distal phalanx angle can be expressed as follows:

(4) $$\begin{array}{c} \frac{\theta_{L}\left(s\right)}{U_{a}\left(s\right)}=\frac{1}{As^{3}+Bs^{2}+Cs}, \end{array}$$

where *A* = *L*_*a*_(*J*_*m*_ *N* + *J*_*L*_)/*K*_*T*_, *B* = [*R*_*a*_(*J*_*m*_ *N* + *J*_*L*_) + *L*_*a*_(*B*_*m*_ *N* + *B*_*L*_)]/*K*_*T*_, and *C* = *R*_*a*_(*B*_*m*_ *N* + *B*_*L*_)/*K*_*T*_ + *NK*_*e*_.

In the single multijoint finger system, the Faulhaber 1319006SR DC servo motor has some important parameters; that is, *B*_*m*_ = 2.22 × 10^−4^ mNm/rpm, *K*_*T*_ = 4.19 mNm/A, *R*_*a*_ = 8.26 Ω, *L*_*a*_ = 130 *μ*H, and *J*_*m*_ = 0.40 gcm^2^. The speed control system consists of a gearbox and one-grade bevel gear, and the gearbox ratio is 415 : 1 and the bevel gears ratio is 2 : 1. Moreover, by using coupling four-bar linkage mechanism, the three phalanxes' transmission ratio is kept exactly 1 : 1 : 1 over the whole movement range. The hand material is ABS; *J*_*L*_ is set to 1 gcm^2^ and *B*_*L*_ is set to 0.002 mNm/rpm. According to the parameters, we can obtain the transfer function as follows:

(5) $$\begin{array}{c} G\left(s\right)=\frac{\theta \left(s\right)}{U_{a}\left(s\right)}=\frac{1}{1.033\times 10^{-6}s^{3}+6.565\times 10^{-2}s^{2}+0.731s}. \end{array}$$

## 4. GA-Fuzzy-Immune PID Controller

### 4.1. Immune-Based PID Controller Design

As a general rule in the discrete-time domain, traditional increment PID controller can be expressed as follows:

(6) u(k)=Kp[e(k)+TTi∑j=0ke(j)+TdTΔe(k)]=u(k−1)+KpΔe(k)+Kie(k) +Kd(Δe(k)−Δe(k−1)),

where Δ*e*(*k*) = *e*(*k*) − *e*(*k* − 1), *K*_*p*_ is the proportional gain, *T*_*i*_ is the integral time constant, *T*_*d*_ is the derivative time constant, *K*_*i*_ = *K*_*p*_ *T*/*T*_*i*_, *K*_*d*_ = *K*_*p*_ *T*_*d*_/*T*, *e*(*k*) is the systematic deviation between reference input and system output, *T* is the sampling period, and *u*(*k*) is the control signal.

In general, differential signal can be used to improve the system dynamic characteristics, which is likely to cause the problem of high frequency interference to the control system. Using low pass filter in control algorithm can bring significant improvements in system performance and its transfer function is *G*_*f*_(*s*) = 1/(1 + *T*_*f*_ *s*), where *T*_*f*_ is a filter coefficient. The transfer function of PID controller with incomplete derivation can be expressed as follows:

(7) U(s)=Kp(1+1Tis+Tds1+Tfs)E(s)=Up+Ui+Ud.

In the discrete-time domain, differential equation of PID controller with incomplete derivation can be written as follows:

(8) $$\begin{array}{c} u\left(k\right)=K_{p}e\left(k\right)+K_{i}\overset{k}{\underset{j=0}{\sum }}e\left(j\right)+u_{d}\left(k\right). \end{array}$$

Then, differentiation element can be expressed as follows:

(9) $$\begin{array}{c} U_{d}\left(s\right)=\frac{K_{p}T_{d}s}{1+T_{f}s}E\left(s\right). \end{array}$$

Thus, we can obtain the differential equation of differentiation element as follows:

(10) $$\begin{array}{c} u_{d}\left(k\right)=K_{d}\left(1-\alpha \right)\left[e\left(k\right)-e\left(k-1\right)\right]+\alpha u_{d}\left(k-1\right), \end{array}$$

where *α* = *T*_*f*_/(*T*_*f*_ + *T*) and *u*_*d*_(0) is the initial value of differentiation element. *α* is set equal to a constant. *α* ^*k*^ is the *k*th power of *α* and *α* ^*k*−*j*^ is the (*k*‒*j*)th power of *α*.

Substituting formula (10) into (8), the PID controller with incomplete derivation can be obtained:

(11) u(k)=Kpe(k)+Ki∑j=1ke(j)+Kd(1−α)[e(k)−e(k−1)] +αud(k−1).

**Figure 3:**
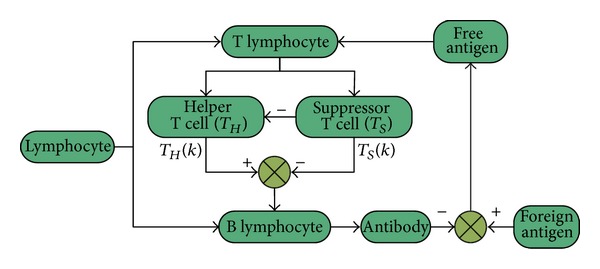
The immunity feedback control mechanism.

As a kind of control system, biological immune system has very strong robustness and self-adapted ability even when encountering strong disturbances and uncertain conditions. For invasion by a foreign antigen, it can produce corresponding antibodies to resist the antigen. A series of biological reactions could be carried out after combining antigens with antibodies and it eliminates antigen under the function of phagocyte or special enzymes. The immune system consists of lymphocyte and antibody. The lymphocyte consists of B cell produced from marrow and T cell produced from thymus. T cell includes assistant T cell *T*_*H*_ and restrained T cell *T*_*S*_. When cell obtains signal from the antigen, it would transmit the information to *T*_*H*_ and *T*_*S*_, and then B cell produces corresponding antibodies to resist the antigen with the stimulation by *T*_*H*_ and *T*_*S*_. The immunity feedback control mechanism is shown in Figure 3.

According to immunity feedback control mechanism, all of the received simulations of B cell can be obtained:

(12) TH(k)=k1ε(k),

(13) Ts(k)=k2f(S(k),ΔS(k))ε(k),

(14) S(k)=TH(k)−TS(k)=k1(1−ηf(S(k),ΔS(k)))ε(k),

where *T*_*H*_(*k*) is the *k*th generation output of *T*_*H*_ cell which receives antigen presenting cell activation, *T*_*S*_(*k*) is the *k*th generation restrain action on B cell by *T*_*S*_ cell, *ε*(*k*) is the *k*th generation antigen amount, *k*_1_ is enhancing factor of *T*_*H*_ cell, *k*_2_ is inhibitory factor of *T*_*S*_ cell, and *η* = *k*_2_/*k*_1_. *f*(∗) is a nonlinear function, which describes the immunity result that B-cell antibody and antigen act on each other and relate with the amount of B cell.

In this paper, we try to apply body's immune mechanism to the ABS-I position controller to overcome the weakness of traditional PID controller. For a PID controller, we assume that position error *e*(*k*) on the *k*th sampling period represents *ε*(*k*); the position controller output *u*(*k*) on the *k*th sampling period represents *S*(*k*). Therefore, Δ*u*(*k*) = Δ*S*(*k*).

In the synthesis, the immune PID controller with incomplete derivation can be obtained:

(15) u(k)=Kp′e(k)+Ki′∑j=1ke(j) +Kd′(1−α)[e(k)−e(k−1)] +αud(k−1),

(16) $$\begin{array}{c} K_{p}^{\prime }=K_{1}\left(1-\eta_{1}f\left(u\left(k\right),\Delta u\left(k\right)\right)\right), \end{array}$$

(17) $$\begin{array}{c} K_{i}^{\prime }=K_{2}\left(1-\eta_{2}f\left(u\left(k\right),\Delta u\left(k\right)\right)\right), \end{array}$$

(18) Kd′=K3(1−η3f(u(k),Δu(k))),

where *K*_*j*_ (*j* = 1,2, 3) is used to improve the response time and *η*_*j*_ (*j* = 1,2, 3) can enhance the stability of control system. Therefore, the method for setting the parameters reasonably plays an important role in the improved PID controller with higher precision, faster response, and better robustness.

### 4.2. Parameters Optimization through Fuzzy Theory and Genetic Algorithm

**Figure 4:**
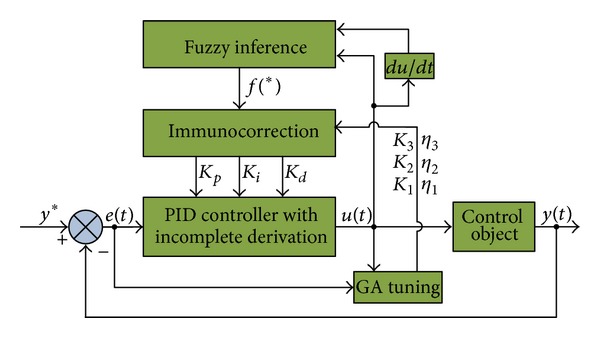
The framework of GA-fuzzy-immune PID position controller with incomplete derivation.

The performance of improved PID controller largely depends on *K*_*j*_ (*j* = 1,2, 3), *η*_*j*_ (*j* = 1,2, 3), and *f*(∗). As can be seen from the above formulas, namely, (15), (16), (17), and (18), because of the nonlinear characteristics of function *f*(∗), a fuzzy inference algorithm is used to optimize the function *f*(∗). Because of the difficulty to obtain *K*_*j*_ (*j* = 1,2, 3) and *η*_*j*_ (*j* = 1,2, 3) based on analysis method, an improved genetic algorithm is proposed to solve this problem. The framework of GA-fuzzy-immune PID position controller with incomplete derivation can be built up as shown in Figure 4.

According to the immune feedback mechanism of biological systems [42], four stages in the autoimmune reaction can be summarized as follows.

In the initial stage, the antigen amount is higher and the antibody amount is expected to increase quickly, so the *T*_*s*_ cell should be suppressed to produce. After a period of immunization, the restrained action on *T*_*s*_ cell would decrease; in other words, the antibody should not increase continually. When most of antigens have been eliminated, *T*_*s*_ should increase quickly to restrain B cell and the production of antibody. Finally, when all of the antigens have been eliminated, both of antigen and antibody amount should keep stable till the immunization end.

In the controller, two inputs of *u*(*k*) and Δ*u*(*k*) fuzzy subsets are all selected as {NB, NS, PS, PB}, and the output of *f*(∗) fuzzy subset is all selected as {NB, NM, NS, ZO, PS, PM, PB}, where NB stands for negative big, NM stands for negative middle, NS stands for negative small, ZO stands for zero, PS stands for positive small, PM stands for positive middle, and PB stands for positive big. According to the above immunologic processes, 16 fuzzy rules are proposed to compute the nonlinear function *f*(∗), as shown in Table 1. The fuzzy discourse domain of *u* is defined as {−10, −3, +3, +10}, the fuzzy discourse domain of Δ*u* is defined as {−1, −0.3, +0.3, +1}, and the fuzzy discourse domain of *f*(∗) is defined as {−2, −1.2, −0.6,0, +0.6, +1.2, +2}.

**Figure 5:**
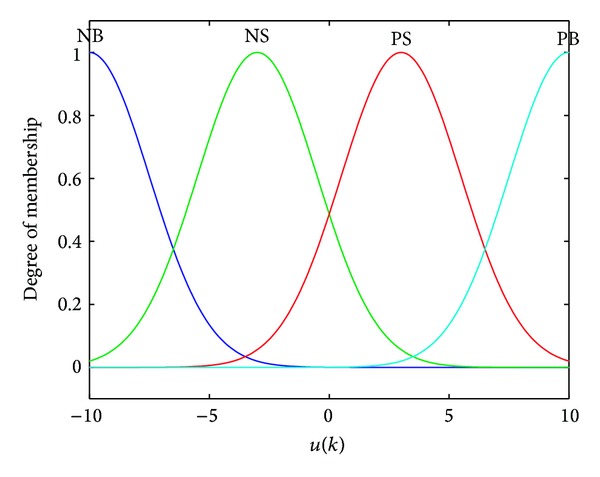
Membership functions for *u*.

**Figure 6:**
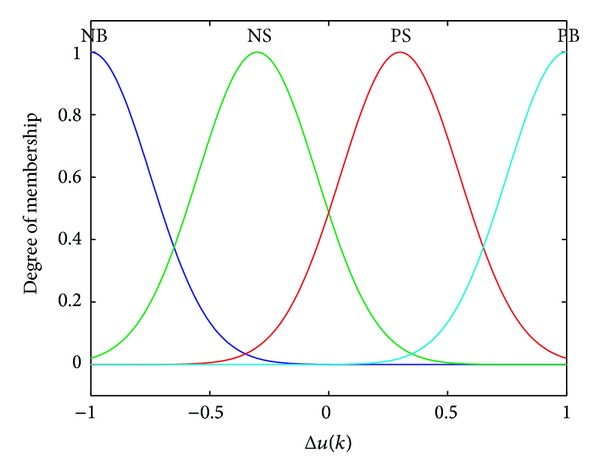
Membership functions for Δ*u*.

**Figure 7:**
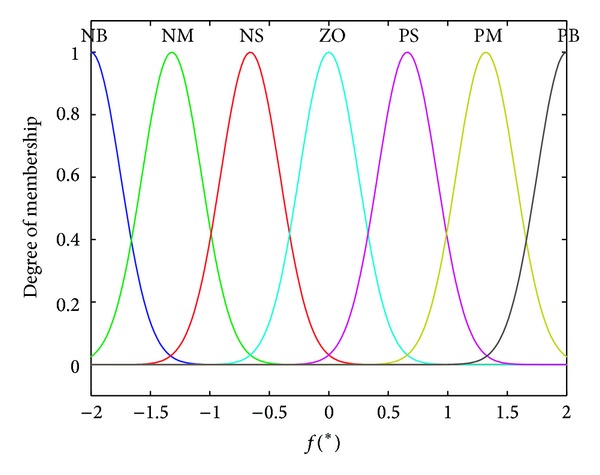
Membership functions for *f*(∗).

As a frequently used membership function, Gaussian membership function has the feature of good smoothness and can express the concept of fuzzy language more exactly; thus, it is applied for the proposed controller.  Figure 5 shows the membership functions for *u*, Figure 6 shows the membership functions for Δ*u*, and Figure 7 shows the membership functions for *f*(∗).

The immune PID parameters *K*_*j*_ (*j* = 1,2, 3) and *η*_*j*_ (*j* = 1,2, 3) are tuned and optimized by an improved genetic algorithm. Traditional genetic algorithm in solving the problem, especially the complex problems, is easily trapped in the local optimum and appeared premature convergence. To settle this question, some improvements of traditional genetic algorithm are presented. The overall process can be described as follows.

**Step 1 (coding).**

As a general coding method for GA, binary coding is used widely due to the simple processes of coding and decoding and easy operation of crossover and mutation. However, for a multivariable optimization problem, the string of binary gene is too long to result in lower search efficiency. In order to solve this problem, float-point genes are used in the optimization model. With this strategy, the number of variables is not limited; coding and decoding are not needed. Furthermore, the precision and efficiency can be increased and the calculation speed is high. A mixed coding program is presented in the improved GA. During the initial stage, binary coding is adopted to quickly search for the area with excellent properties. In the later stage, float-point coding is used to improve the precision.

**Step 2 (generating initial population).**

According to experience, six empirical coefficients (*K*_1_,  *K*_2_,  *K*_3_,  *η*_1_,  *η*_2_ and *η*_3_) are determined and initial population can be generated around the coefficients. By this generating method, the searching space is reduced and the operating rate is increased.

**Step 3 (selecting fitness function).**

In an evolution search process, an appropriate fitness function plays an important role in parameter optimization. In order to obtain satisfactory dynamic characteristics of the transition process, the integral of time multiplied absolute value of error (ITAE) is also provided as a comprehensive performance index, and the square of control input is introduced to prevent the control energy from growing too big. The comprehensive performance index function [43] can be calculated as follows:

(19) $$\begin{array}{c} J=\{\begin{array}{cc} \overset{\infty }{\underset{0}{\int }}\left(\omega_{1}\left|e\left(t\right)\right|+\omega_{2}u^{2}\left(t\right)\right)dt & \\ \;+\omega_{3}t_{r} & e\left(t\right)\geq 0,\\ \overset{\infty }{\underset{0}{\int }}\left(\omega_{1}\left|e\left(t\right)\right|+\omega_{2}u^{2}\left(t\right)+\omega_{4}\left|e\left(t\right)\right|\right)dt & \\ \;+\omega_{3}t_{r} & e\left(t\right)<0, \end{array} \end{array}$$

where *ω*_1_, *ω*_2_, *ω*_3_, and *ω*_4_ are weights and *ω*_4_ ≫ *ω*_1_, *e*(*t*) is the system error, *u*(*t*) is the output of controller, and *t*_*r*_ is the rising time. To avoid overshoot, the introduction of punitive function is essential in the function.

Then, the fitness function *F* can be defined as follows:

(20) $$\begin{array}{c} F=\frac{C}{\left(J+\varepsilon \right)}, \end{array}$$

where *C* is a constant and can be set equal to 1 in this paper, *ε* is a small positive number to prevent *J* from becoming equal to zero, and *ε* = 10^−10^.

**Step 4 (selection).**

Selection is a very important step in the criteria of “survival of the fittest” that means selecting the superior individual and eliminating the inferior one from a population. For genetic algorithm, an individual is selected as a parent according to its fitness. In rank-based selection algorithm, all individuals of every generation are ranked in order of increasing fitness value. The survival probability of the *i*th individual is prob(*i*) = *q*(1−*q*)^*i*−1^, where *q* ∈ (0,1) is evolutionary pressure.

**Step 5 (crossover and mutation).**

Because of its strong global search capability, crossover operator of GA can be regarded as the main operator, and due to its local search capability, mutation operator can be regarded as an auxiliary operator. Self-adaptive crossover and mutation operators are proposed in this paper; in other words, crossover probabilities *P*_*c*_ and mutation probabilities *P*_*m*_ are automatically adjusted with the addition of evolutionary generations. In the initial stage, a larger *P*_*c*_ and a smaller *P*_*m*_ can effectively accelerate convergence velocity of iteration; however, in the later stage, a smaller *P*_*c*_ and a larger *P*_*m*_ would avoid local optimal solution. The formulas of *P*_*c*_ and *P*_*m*_ are given as follows:

(21) $$\begin{array}{c} P_{c}\left(k+1\right)=P_{c}\left(k\right)-\frac{\left[P_{c}\left(1\right)-0.5\right]}{G_{m}}, \end{array}$$

(22) $$\begin{array}{c} P_{m}\left(k+1\right)=P_{m}\left(k\right)-\frac{\left[P_{m}\left(1\right)-0.1\right]}{G_{m}}, \end{array}$$

where *k* is the generation number of heredity, *k* = 1 ~ *G*_*m*_, *G*_*m*_ is the maximum generation number, *P*_*c*_(1) is the crossover probability of first generation, and *P*_*m*_(1) is the mutation probability of first generation.

According to these operators, the *P*_*c*_ and *P*_*m*_ of best individuals are not equal to zero, where *P*_*c*_ ∈ (0.5, *P*_*c*_(1)) and *P*_*m*_ ∈ (*P*_*m*_(1), 0.1), so the performance of excellent individual would not be in a circle due to the *P*_*c*_ and *P*_*m*_ being too small or equal to zero. To protect excellent individuals of each generation, the elitist strategy was applied in GA to improve the convergence and optimization results; thus, the best individual would be copied directly into next generation.

## 5. A Simulation Example

In order to verify the performance of proposed GA-fuzzy-immune PID controller, a simulation example is provided in this section and the parameters are illustrated as follows.

*ω*_1_ = 0.04, *ω*_2_ = 0.001, *ω*_3_ = 2, and *ω*_4_ = 500. The population size is set to 50, *G*_*m*_ is set to 100, *P*_*c*_(1) is set to 0.9, *P*_*m*_(1) is set to 0.01, *T*_*f*_ is set to 9, and sampling time *T* is set to 1 ms.

In order to indicate the comparison with other controllers, fuzzy PID, immune PID, fuzzy-immune PID, and real-coded GA PID simulations are carried out. The configurations of simulation environment for these controllers were uniform. In immune PID and fuzzy-immune PID, *K*_1_ = 10, *K*_2_ = 0.02, *K*_3_ = 1.0, *η*_1_ = 0.02, *η*_2_ = 0.06, and *η*_3_ = 1.0, and *f*(∗) = 0.01 in immune PID. In fuzzy PID and real-coded GA PID, *K*_*p*_ ∈ (0,80), *K*_*i*_ ∈ (0,2), and *K*_*d*_ ∈ (0,2). Other parameters are the same as GA-fuzzy-immune PID.

**Figure 8:**
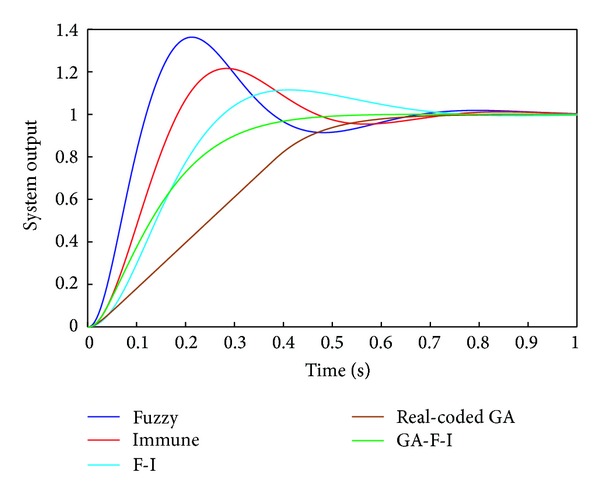
Unit step responses of system.

The input of robot dexterous hand system is a unit step signal and the simulation time is 1 s. The unit step responses of this system are shown in Figure 8. The first curve is response obtained with fuzzy inference, the second curve is response obtained with immune algorithm, the third curve is response obtained with fuzzy-immune inference (F-I), the fourth curve is response obtained with real-coded GA, and the fifth curve is response obtained through integration of improved genetic algorithm and fuzzy-immune inference (GA-F-I).

The PID parameters and performance indexes of the five control methods are shown in Table 2. The proposed controller parameters can be calculated by improved GA and fuzzy inference:

(23) $$\begin{array}{c} K_{1}=11.14655,\;\;\;K_{2}=0.01737,\;\;\;K_{3}=0.81208,\\ \eta_{1}=0.02121,\;\;\eta_{2}=0.06604,\;\;\eta_{3}=0.94233,\\ f\left(*\right)=0.000544. \end{array}$$

Compared with other four methods, the overshoot *σ*  based on GA-F-I PID controller with incomplete derivation is decreased from 36.30% to 0. The settling time *t*_*s*_ is reduced from 0.592 s to 0.362 s. The rising time *t*_*r*_ is reduced from 0.393 s to 0.226 s. Although the rising time *t*_*r*_ is not the best, the nonovershoot and shortest settling time can be achieved by the proposed PID controller.

## 6. Conclusions and Future Works

In this paper, a GA-fuzzy-immune PID controller was designed to improve the performance of robot dexterous hand. The control system of a robot dexterous hand and mathematical model of an index finger were presented. In order to improve the characteristics of proposed controller, immune mechanism, genetic algorithm, and fuzzy inference were applied. Finally, a simulation experiment was carried out and the results showed that the designed controller was ideal.

In future studies, the authors plan to investigate multifinger coordination control system. Furthermore, more intelligent control algorithms for multifinger coordination control system are worth further study for the authors.

## Figures and Tables

**Table 1:** The fuzzy control rule for nonlinear function *f*(∗).

*u*	Δ*u*			
	NB	NS	PS	PB
NB	PB	PM	PS	ZO
NS	PM	PS	ZO	NS
PS	PS	ZO	NS	NM
PB	ZO	NS	NM	NB

**Table 2:** PID parameters and performance indexes of five control methods.

Control methods	Fuzzy	Immune	F-I	Real-coded GA	GA-F-I
*K*_*p*_	8.152	9.998	4.996	60.345	11.146
*K*_*i*_	0.840	0.020	0.101	1.896	0.017
*K*_*d*_	0.209	0.050	0.100	0.021	0.812
*σ*/%	36.30	21.61	11.54	0	0
*t*_*s*_/s	0.578	0.426	0.592	0.521	0.362
*t*_*r*_/s	0.079	0.105	0.182	0.393	0.226

## References

[1] Bicchi A, Kumar V Robotic grasping and contact: a review.

[2] Liu H, Meusel P, Seitz N (2007). The modular multisensory DLR-HIT-hand. *Mechanism and Machine Theory*.

[3] Controzzi M, Cipriani C, Jehenne B, Donati M, Carrozza MC Bio-inspired mechanical design of a tendon-driven dexterous prosthetic hand.

[4] Murray RM, Sastry SS (1994). *A Mathematical Introduction to Robotic Manipulation*.

[5] Yoshikawa T (2010). Multifingered robot hands: Control for grasping and manipulation. *Annual Reviews in Control*.

[6] Tomovic R, Boni G (1962). An adaptive artificial hand. *Transactions on Automatic Control, IRE*.

[7] Jafarov EM, Istefanopulos Y, Parlakçi MNA (2002). A new variable structure PID-controller for robot manipulators with parameter perturbations: an augmented sliding surface approach. *Sign*.

[8] Jafarov EM, Parlakçi MNA, Istefanopulos Y (2005). A new variable structure PID-controller design for robot manipulators. *IEEE Transactions on Control Systems Technology*.

[9] Atia KR (2013). A new variable structure controller for robot manipulators with a nonlinear PID sliding surface. *Robotica*.

[10] Chen Y, Ma G, Lin S, Gao J (2012). Adaptive fuzzy computed-torque control for robot manipulator with uncertain dynamics. *International Journal of Advanced Robotic Systems*.

[11] Park SH, Han SI (2011). Robust-tracking control for robot manipulator with deadzone and friction using backstepping and RFNN controller. *IET Control Theory & Applications*.

[12] Hassanzadeh I, Alizadeh G, Hashemzadeh F (2011). Performance tuning for robot manipulators using intelligent robust controller. *Proceedings of the Institution of Mechanical Engineers, Part I: Journal of Systems and Control Engineering*.

[13] Siqueira AAG, Terra MH, Buosi C (2007). Fault-tolerant robot manipulators based on output-feedback H_∞_ controllers. *Robotics and Autonomous Systems*.

[14] Han JQ (2009). From PID to active disturbance rejection control. *IEEE Transactions on Industrial Electronics*.

[15] Su YX, Duan BY, Zheng CH (2004). Nonlinear PID control of a six-DOF parallel manipulator. *IEE Proceedings: Control Theory and Applications*.

[16] Gundes AN, Ozguler AB (2007). P{ID} stabilization of {MIMO} plants. *IEEE Transactions on Automatic Control*.

[17] Alvarez-Ramirez J, Kelly R, Cervantes I (2003). Semiglobal stability of saturated linear PID control for robot manipulators. *Automatica*.

[18] Oliveira VA, Cossi LV, Teixeira MCM, Silva AMF (2009). Synthesis of PID controllers for a class of time delay systems. *Automatica*.

[19] Ziegler JG, Nichols NB (1942). Optimum setting for automatic controllers. *ASME Transactions*.

[20] Chen J, Huang T (2004). Applying neural networks to on-line updated PID controllers for nonlinear process control. *Journal of Process Control*.

[21] Pelusi D Genetic-neuro-fuzzy controllers for second order control systems.

[22] Pelusi D On designing optimal control systems through genetic and neuro-fuzzy techniques.

[23] Pelusi D (2012). PID and intelligent controllers for optimal timing performances of industrial actuators. *International Journal of Simulation: Systems, Science and Technology*.

[24] Pelusi D, Vazquez L, Diaz D Fuzzy algorithm control effectiveness on drum boiler simulated dynamics.

[25] Pelusi D Improving settling and rise times of controllers via intelligent algorithms.

[26] Pelusi D (2013). Designing neural networks to improve timing performances of intelligent controllers. *Journal of Discrete Mathematical Sciences and Cryptography*.

[27] Pelusi D, Mascella R (2013). Optimal control algorithms for second order systems. *Journal of Computer Science*.

[28] Kim T, Maruta I, Sugie T (2008). Robust PID controller tuning based on the constrained particle swarm optimization. *Automatica*.

[29] Juang CF, Lu CF (2006). Load-frequency control by hybrid evolutionary fuzzy PI controller. *IEE Proceedings: Generation, Transmission & Distribution*.

[30] Lu C, Hsu C, Juang C (2013). Coordinated control of flexible AC transmission system devices using an evolutionary fuzzy lead-lag controller with advanced continuous ant colony optimization. *IEEE Transactions on Power Systems*.

[31] Tang KS, Man KF, Chen G, Kwong S (2001). An optimal fuzzy PID controller. *IEEE Transactions on Industrial Electronics*.

[32] Zheng F, Wang Q, Lee TH (2002). On the design of multivariable PID controllers via LMI approach. *Automatica*.

[33] Farmer JD, Packard NH, Perelson AS (1986). The immune system, adaptation, and machine learning. *Physica D: Nonlinear Phenomena*.

[34] Xin J, Liu D, Yang Y Robot trajectory tracking control based on fuzzy immune PD-type controller.

[35] Lei Z, Ren-hou L (2008). Designing of classifiers based on immune principles and fuzzy rules. *Information Sciences*.

[36] Wang SX, Jiang Y, Yang H Chaos optimization strategy on fuzzy-immune-PID control of the turbine governing system.

[37] Lin WB, Chiang HK, Chung YL (2013). The speed control of immune-fuzzy sliding mode controller for a synchronous reluctance motor. *Applied Mechanics and Materials*.

[38] Chang GW, Chang W, Chuang C, Shih D (2011). Fuzzy logic and immune-based algorithm for placement and sizing of shunt capacitor banks in a distorted power network. *IEEE Transactions on Power Delivery*.

[39] Kuo RJ, Tseng WL, Tien FC, Warren Liao T (2012). Application of an artificial immune system-based fuzzy neural network to a RFID-based positioning system. *Computers and Industrial Engineering*.

[40] Lianjie E (1994). *Automatic Control System*.

[41] Er G, Dou Y (2002). *Motion Control System*.

[42] Deng J, Li XY, Wei W (2011). OPC controller for turbo-generating set based on immune fuzzy algorithm. *Proceeding of the CSU-EPSA*.

[43] Liu JK (2011). *Advanced PID Control based on MATLAB*.

